# The synergistic effects of PRDX5 and Nrf2 on lung cancer progression and drug resistance under oxidative stress in the zebrafish models

**DOI:** 10.32604/or.2022.026302

**Published:** 2023-01-05

**Authors:** SITONG QIAN, YING FANG, CHENGYUN YAO, YONGSHENG WANG, ZHI ZHANG, XIAOHUA WANG, JIN GAO, YONG FENG, LEI SUN, RUNYUE ZOU, GUOREN ZHOU, JINJUN YE, RUIXUE XIA, HONGPING XIA

**Affiliations:** 1 Jiangsu Cancer Hospital, The Affiliated Cancer Hospital of Nanjing Medical University, Jiangsu Institute of Cancer Research, Nanjing, 210009, China; 2 School of Life Sciences, Nanjing Normal University, Nanjing, 210046, China; 3 Department of Respiratory Medicine, Nanjing Drum Tower Hospital Affiliated to Medical School of Nanjing University, Nanjing, 210008, China; 4 Medical College of Henan University & Henan University Huaihe Hospital, Kaifeng, 475000, China; 5 Zhongda Hospital, School of Medicine & Advanced Institute for Life and Health, Southeast University, Nanjing, 210009, China; 6 Department of Pathology, Nanjing Drum Tower Hospital & Drum Tower Clinical College & School of Basic Medical Sciences & Key Laboratory of Antibody Technique of National Health Commission & Jiangsu Antibody Drug Engineering Research Center, Nanjing Medical University, Nanjing, 211166, China

**Keywords:** PRDX5, Nrf2, Oxidative stress, Lung cancer, Drug resistance, Zebrafish models

## Abstract

Previous studies have shown that PRDX5 and Nrf2 are antioxidant proteins related to abnormal reactive oxidative species (ROS). PRDX5 and Nrf2 play a critical role in the progression of inflammations and tumors. The combination of PRDX5 and Nrf2 was examined by Co-immunoprecipitation, western blotting and Immunohistochemistry. H_2_O_2_ was applied to affect the production of ROS and induced multi-resistant protein 1 (MRP1) expression in NSCLC cells. The zebrafish models mainly investigated the synergistic effects of PRDX5 and Nrf2 on lung cancer drug resistance under oxidative stress. We showed that PRDX5 and Nrf2 form a complex and significantly increase the NSCLC tissues compared to adjacent tissues. The oxidative stress improved the combination of PRDX5 and Nrf2. We demonstrated that the synergy between PRDX5 and Nrf2 is positively related to the proliferation and drug resistance of NSCLC cells in the zebrafish models. In conclusion, our data indicated that PRDX5 could bind to Nrf2 and has a synergistic effect with Nrf2. Meanwhile, in the zebrafish models, PRDX5 and Nrf2 have significant regulatory impacts on lung cancer progression and drug resistance activities under oxidative stress.

## Introduction

Non-small cell lung cancer (NSCLC) is the most common type of lung cancer, with the highest mortality rate among malignant tumors in the world [1]. The 5-year survival rate of NSCLC patients is still less than 15% [2]. Therefore, exploring the mechanism of NSCLC occurrence and progression is crucial. Reactive oxygen species (ROS) is the most dominant part of oxidative stress and is highly associated with cancer [3,4]. Abnormal generation of ROS can cause tumor resistance and metastasis [5] or act as a second messenger in tumorigenesis and development [6]. Therefore, it is essential to maintain the homeostasis of ROS in the body through the antioxidant system that removes different types of ROS. Peroxiredoxins (PRXs) is a member of the antioxidant system with six members PRDX1-6 [7]. They are ideal ROS scavengers. PRX contains cysteine residues sensitive to oxidation, which helps PRX reduce H_2_O_2_ to H_2_O [8]. H_2_O_2_ can regulate the expression of tumor suppressor genes and participate in the proliferation and migration of cancer cells [9].

In mammals, PRDX5 targeted to the mitochondrial matrix [8] and mitochondria constitute a significant source of H_2_O_2_ [8]. Many types of malignant tumors can observe the abnormal expression of PRDX5 [10]. PRDX5 relates to cell proliferation, differentiation, and cell signal transduction [11,12], and is closely associated with tumor size, lymphatic invasion, and drug resistance [13,14]. PRDX5 overexpression in mitochondria can prevent DNA damage caused by peroxides and apoptosis induced by P53 [15]. In contrast, cells lacking PRDX5 have increased protein carboxyl levels, DNA damage, and enhanced sensitivity to H_2_O_2_ [16].

Nuclear respiratory factor 2 (Nrf2; GABPA) is a transcription factor that has been shown to regulate the expression of Prdx5 in response to oxidative stress and mitochondrial biogenesis [17,18]. The Nrf2-ARE signaling pathway is an essential negative regulatory system that reduces the body’s ROS content. Under normal physiological conditions, Nrf2 in the Nrf2-ARE pathway binds to the negative regulator Keap1 to maintain the continuous degradation of Nrf2 and maintain a low level of Nrf2 expression in cells [19]. When ROS increases, the conformation of Keap1 is changed to release Nrf2, which makes Nrf2 out of the ubiquitination and degradation state. Then Nrf2 enters the nucleus, binds to downstream genes’ antioxidant response element (ARE), and regulates antioxidant target genes [20]. Many studies have shown that under the action of ROS, the abnormal expression of Nrf2 can induce lung cancer, promote the proliferation of cancer cells and secretion of drug-resistant proteins, and inhibit p53 and Bcl-2 mediated apoptosis [21–25]. Nrf2 is activated in lung cancer cells to increase the production of antioxidant proteins and maintain redox balance [7]. However, the regulation of Nrf2 concentration by way of Keap1 mutations is only 3% to 5% [26]. Therefore, finding other pathways that can regulate Nrf2 expression is imperative.

Xinming Chen’s study has discussed in detail that PRDX5 is a novel binding protein for Nrf2 and demonstrated the mechanism of their interaction under oxidative stress conditions, which will not be discussed in detail in this paper [27]. Some scholars have indicated that myeloid-derived cancer cells can promote tumor metastasis by downregulating Nrf2 expression [28,29]. Moreover, several studies on drug resistance in lung cancer suggest that the homeostatic maintenance of Nrf2 with ROS endogenous seems crucial in the expression of multidrug resistance protein 1 (MRP1) [30–32]. MRP1 belongs to the ATP-binding cassette (ABC) transporter protein superfamily and is a key mechanism contributing to the poor outcome of cancer chemotherapy [33]. MRP1 acts as a transmembrane protein that uses the energy of ATP hydrolysis to exclude drugs and prevent intracellular drug accumulation, resulting in the inability of many chemotherapeutic drugs (e.g., cisplatin) to effectively inhibit the proliferation and metastasis of cancer cells [32]. Therefore, we speculate that the interaction mechanism between PRDX5 and Nrf2 may also link to drug resistance in NSCLC.

In this study, we applied the zebrafish model for the first time to the effects of PRDX5 and Nrf2 interaction mechanisms on NSCLC proliferation, metastasis, and drug resistance. Zebrafish *(Danio rerio)*, an animal model with 87% genetic homology to humans, has been widely used for disease research and drug development. The zebrafish model is highly visual and can mimic the clinical tumor microenvironment and has been used in several cancer studies [34,35]. In gastric cancer studies, zebrafish xenograft models exhibited highly similar drug sensitivity and phenotypes to clinical patients [36]. Jiang Ren’s research suggests that the zebrafish xenograft model can also be used to track the progression of breast cancer diseases, such as cancer cell invasion and metastasis [37]. RuoYue Fan explored the mechanism of NSCLC brain metastasis and rescue strategies using a zebrafish xenograft model [38].

In this study, we used a zebrafish xenograft model to visualize the biological behavior of NSCLC cells in terms of proliferation, metastasis, and drug resistance concerning the interaction mechanism between PRDX5 and Nrf2 under ROS. This study furthers understanding of the critical role of PRDX5-Nrf2 and provides potential diagnostic and therapeutic targets for the treatment of NSCLC.

## Materials and Methods

### Patients and clinical samples

The NSCLC tissue samples were collected from Jiangsu Cancer Hospital. Paired samples of the NSCLC tissues and adjacent tissues were obtained by surgery. After surgical removal, the tissues were collected and immediately frozen in liquid nitrogen and stored at −80°C. This study was approved by the Ethics Committee of Jiangsu Cancer Hospital. All patients have obtained prior written informed consent and approval.

### Cell culture

NSCLC cell lines A549 and H1299 provided by the Jiangsu Institute of Cancer Research. A549 and H1299 were cultured in RPMI 1640 medium supplemented with 10% FBS and 1% Penicillin-Streptomycin. Both of these two cell lines were cultured in a humidified atmosphere containing 5% CO_2_ at 37°C.

### Transfection

A549 and H1299 were seeded in a 24-well plate. When cells confluence reached 70%, the plasmids (pcDNA3.1-PRDX5/Flag, pHBLV-U6-shRNA-PRDX5-ZsGreen, HBLB-h-shRNA-Nrf2-GFP-PURO) were mixed with Lipofectamine 2000 (Invitrogen, USA) in Opti-MEM and incubate for 20 min at room temperature. The DNA mixture was added to cell wells. Then the cells were incubated for 24 h before changing the new medium.

### Co-immunoprecipitation assay

Cells or tumor tissues were washed with PBS, harvested, and lysed with IP lysate mixed with PMSF, cocktail, and DTT at 4°C for 30 min. Then the mixture was centrifuged at 12000 rpm for 15 min at 4°C. 5% protein solution in each group was taken as input which was added 20 ul protein A+G in. Removed agarose beads after preincubating for 2 h at 4°C. Each group of samples was divided into two equal parts. One was added to the target protein IP antibody, and the other was added to an equal amount of IgG isotype control, then incubated overnight at 4°C. Added 20 ul of protein A+G to each tube and set at 4°C for 2 h to bind the antibody to form a protein complex. Removed the supernatant by centrifugation, and the agarose beads were washed 5 times with IP lysate for 5 min each time. Boiled for 10 min, then took 20 ul sample for Western blot test.

### Immunoblot analysis

For immunoblot analysis, cells were washed with phosphate-buffered saline (PBS), harvested, and lysed with RIPA buffer at 4°C for 30 min. Proteins were separated on 8%~12% sodium dodecyl sulfate-polyacrylamide gels and transblotted onto a PVDF membrane by electroblotting. Membranes were incubated with the appropriate primary antibody and HRP-conjugated anti-rabbit antibody. The immunoreactive bands were visualized using the enhanced chemiluminescence (ECL) (Beyotime, Shanghai, China) detection kit.

### Immunohistochemistry

Fix the tissue with paraffin, then cut the wax block into slices (4~8 μm). After the sections were deparaffinized to water, the 0.01 M citrate buffer (pH 6.0) was used for antigen retrieval. After blocking with goat serum, add 1:100 primary antibody dilution and incubate overnight at 4°C. After setting the sections with a horseradish peroxidase-labeled secondary antibody at 37°C for 2 h, DAB was used for color development. Observe the degree of staining of the section under a microscope (a positive result is brown particle precipitation).

### RT-QPCR analysis

According to the manufacturer’s instructions, the total RNA was isolated from A549 and H1299 cells using a TRIpure reagent kit (Aidlab Biotechnologies, Beijing, China). The RNA (500 μg) was reversibly transcribed with 4 μl 4× gDNA wiper Mix, 1 μl Oligo (dT) 18 (10 μM) primer, and 4 μl 5×HiScript® II Select qRTSuperMix II (Vazyme Biotech, Nanjing, China). Then we used the 2×AceQ qPCR SYBR Green Master Mix (Vazyme Biotech, Nanjing, China) and specific primers to perform PCR amplification. The following forward and reverse primers (5′-3′) were used: *GAPDH* forward, AGA TCC CTC CAA AAT CAA GTG G; and *GAPDH* reverse, GGC AGA GAT GAT GAC CCT TTT; *MRP1* forward, TCT CAA CAA AAC CAA AAC TGC CT; and *MRP1* reverse, CTG AAC TCC CTT CCT CCT CTC C. Observing the specificity of the melting curve after the reaction and calculating the 2^−ΔΔCt^ according to the formula. The experiment was repeated three times.

### 4-(4,5-dimethylthiazol-2-yl)-2,5-diphenyltetrazolium bromide (MTT) assay

A549 and H1299 cells were seeded in 96-well plates, cultured for 24 h, and then fed with fresh medium for cisplatin treatment. We set six treatment concentrations of cisplatin (0, 12.5, 25, 50, 100, 200) μg/ml to stimulate cells for 24 h. Removed the medium, added the MTT (0.5 mg/ml) to each well and then incubated the cells for 4 h. Melting purple formazan crystals with DMSO, then measuring it at 490 nm with a microplate reader (BioTek, USA).

### Cell labeling, xenograft and enumeration procedure

Cell line H1299 was labeled with either CM-Dil (red fluorescence) (Invitrogen, USA) or DiO (green fluorescence) (Beyotime, Shanghai, China). We evaluated cell viability with trypan blue staining before injection. The single-cell suspension was formulated into the certain density of 2 × 10^7^ cells per milliliter. Then we used 1 mg/ml of pronase (Sigma-Aldrich, USA) to remove the chorionic membrane of 24 h post-fertilization (hpf) zebrafish embryos and put them in a medium containing 0.2 mM 1-phenyl 2-thiourea (PTU), incubated at 28.5°C for 24 h.

At 48 hpf, the embryos were anesthetized with 0.003% tricaine (Sigma-Aldrich, USA) and placed on a moist agarose plate in a lying position. According to the purpose of the experiment, the single-cell suspension was injected into the yolk sac or perivitelline space of the zebrafish embryo by microinjection (IM-31, Japan).

A group of 10 embryos was sacrificed and dissociated into a single-cell suspension. The number of CM-DiI-labeled cells was enumerated to be the baseline number of H1299 cells before treatment with a vehicle or drug to ensure cells engraft and proliferate in the zebrafish embryos [39]. In this study, we executed the embryos according to the protocol mentioned above on the first and third days after injection and counted the dissociated cells.

### Microscope imaging

We photographed the embryo’s proliferation and migration of H1299 cells on the 1st and 4th day after the injection (dpi) by an inverted fluorescent microscope (ZEISS, Germany).

### Statistical analysis

The results were analyzed and expressed as the mean ± SEM from at least three independent experiments. Comparisons were made using ANOVA and the Student’s test. Differences were considered to be significant at *p* < 0.05.

## Results

### PRDX5 and Nrf2 form a complex and are highly expressed in NSCLC tissues

**Figure 1:**
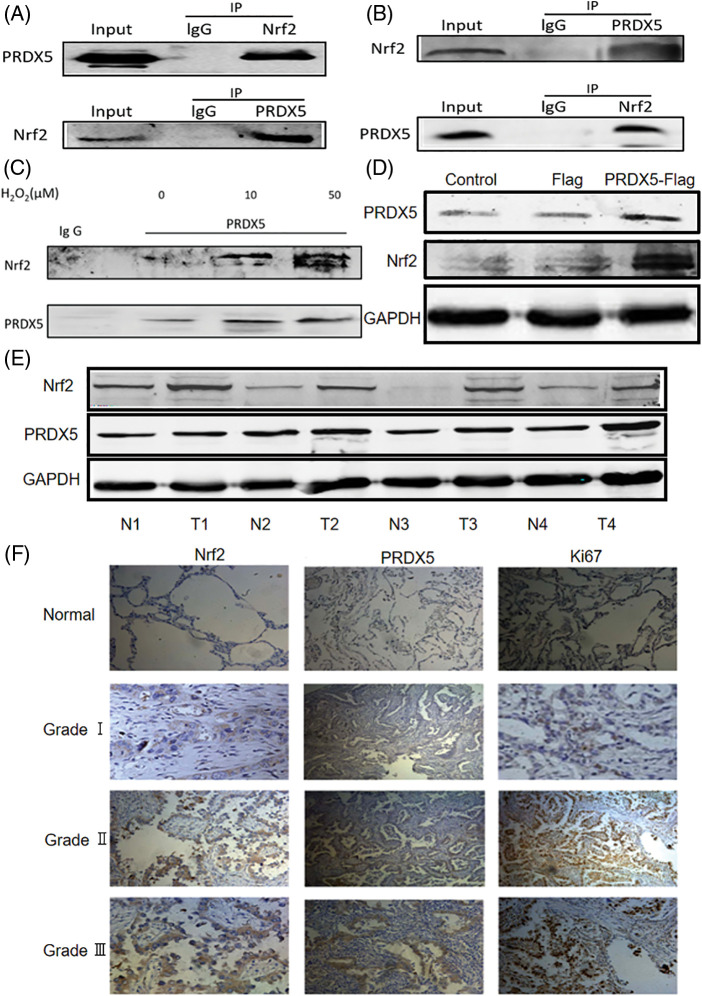
PRDX5 and Nrf2 form the complex and are abnormally expressed in NSCLC tissues. (A) The co-immunoprecipitation assay detected whether PRDX5 and Nrf2 could form a complex in H1299 cells. (B) The co-immunoprecipitation assay detected whether PRDX5 and Nrf2 could form a complex in NSCLC tissues. (C) We stimulated H1299 cells with 0, 10, and 50 μM H_2_O_2_ for 30 min and then extracted cell proteins. The co-immunoprecipitation assay detected changes in the binding degree of PRDX5 and Nrf2. (D) We constructed a PRDX5-Flag overexpression vector and transfected the vector into H1299 cells, which were used to detect the changes in PRDX5 and Nrf2 expression. (E) Western blotting method was used to detect the protein expression of PRDX5 and Nrf2 in NSCLC tissues and paired adjacent tissues. GAPDH was measured as a loading control. T stands for Tumor tissue, and N stands for adjacent tissues. (F) Immunohistochemistry was used to detect the expression of PRDX5, Nrf2, and Ki67 in NSCLC tissues (c~k) and adjacent normal tissues (a~c); comparisons were made according to the grade of malignancy. Scale bar, 100 μm.

We used a co-immunoprecipitation assay to test whether PRDX5 and Nrf2 can combine to form a complex. Fig. 1A showed that PRDX5 and Nrf2 in NSCLC cell proteins could combine to form a complex. At the same time, in the tissue protein of NSCLC, we also found that PRDX5 and Nrf2 could form a complex (Fig. 1B). In addition, under the stimulation of H_2_O_2_, the binding strength was enhanced with the increase of oxidative stress intensity (Fig. 1C). Besides, we observed that after PRDX5 overexpressed in NSCLC cells, Nrf2 expression also up-regulated (Fig. 1D).

We took surgical tissue samples for verification to prove that PRDX5 and Nrf2 were abnormally expressed in the pathogenesis of NSCLC. The results showed that PRDX5 and Nrf2 were highly expressed in NSCLC tissues compared with the corresponding adjacent tissues (Fig. 1E). Furthermore, immunohistochemistry results showed that Nrf2, PRDX5 and Ki67 were not expressed or under-expressed in normal tissues (Fig. 1F). We also found that the higher the tumor grade, the stronger the positive staining signal.

### H_2_O_2_ affected the production of ROS and induced multi-resistant protein 1 (MRP1) expression in NSCLC cells

**Figure 2:**
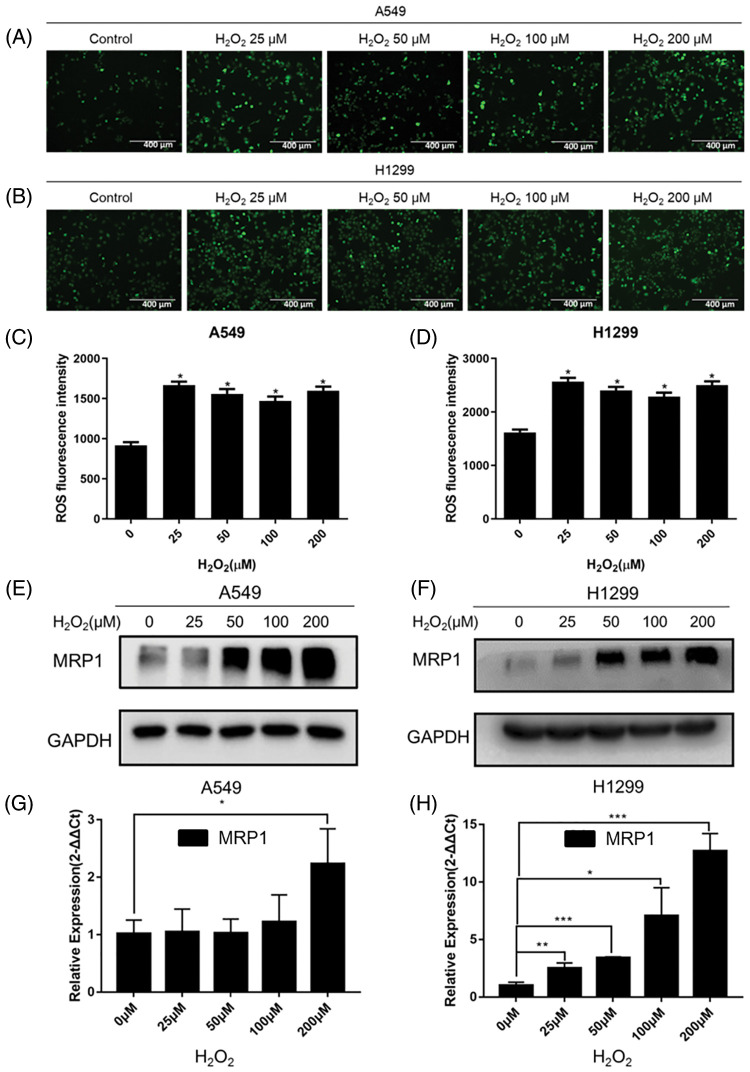
H_2_O_2_ affected the content of ROS and induced MRP1 expression in NSCLC cells. (A–B) Different concentrations (0, 25, 50, 100, 200) μM H_2_O_2_ were used to stimulate A549 (A) and H1299 (B) cells for 24 h. DCFH-DA fluorescent probe was used to detect the content of ROS. The 0 μM was regarded as the control group. Scale bar, 400 μm. (C–D) Statistical analysis of green fluorescence intensity. *p < 0.05. (E–F) The A549 and H1299 cells were treated with different concentrations (0, 25, 50, 100, 200 μM) of H_2_O_2_ for 24 h. We used western blot to detect the expression of MRP1. GAPDH was measured as a loading control. (G–H) The A549 and H1299 cells were treated with different concentrations (0, 25, 50, 100, 200 μM) of H_2_O_2_ for 24 h. Total RNA was isolated from these cells, and then specific primers (MRP1) were used to perform RT-QPCR amplification. GAPDH was used as the reference gene. The experimental results were expressed as mean ± SD. The experiment was repeated three times, *p < 0.05, **p < 0.01, and ***p < 0.001.

To analyze the effect of H_2_O_2_ on the production of ROS in NSCLC cells, we used a DCFH-DA fluorescent probe to detect ROS content in NSCLC cells. The results showed that the content of ROS significantly increased in A549 and H1299 cells under the stimulation of H_2_O_2_ (Figs. 2A–2D). Oxidative stress is a cascade activation response caused by the abnormal production of reactive oxygen species. Our results demonstrated that under the stimulation of H_2_O_2_, A549 and H1299 cells had entered a state of oxidative stress where ROS accumulation was more significant than clearance. Next, we tested the effect of H_2_O_2_ on the expression of MRP1 in NSCLC cells. As shown in Figs. 2E–2H, the results indicated that H_2_O_2_ could induce a concentration-dependent up-regulation of MRP1 in A549 and H1299 cells, both at the protein and mRNA levels.

### The synergistic effect of PRDX5 and Nrf2 regulates the expression of MRP1 under oxidative stress and contributes to the resistance of NSCLC cells to cisplatin

**Figure 3:**
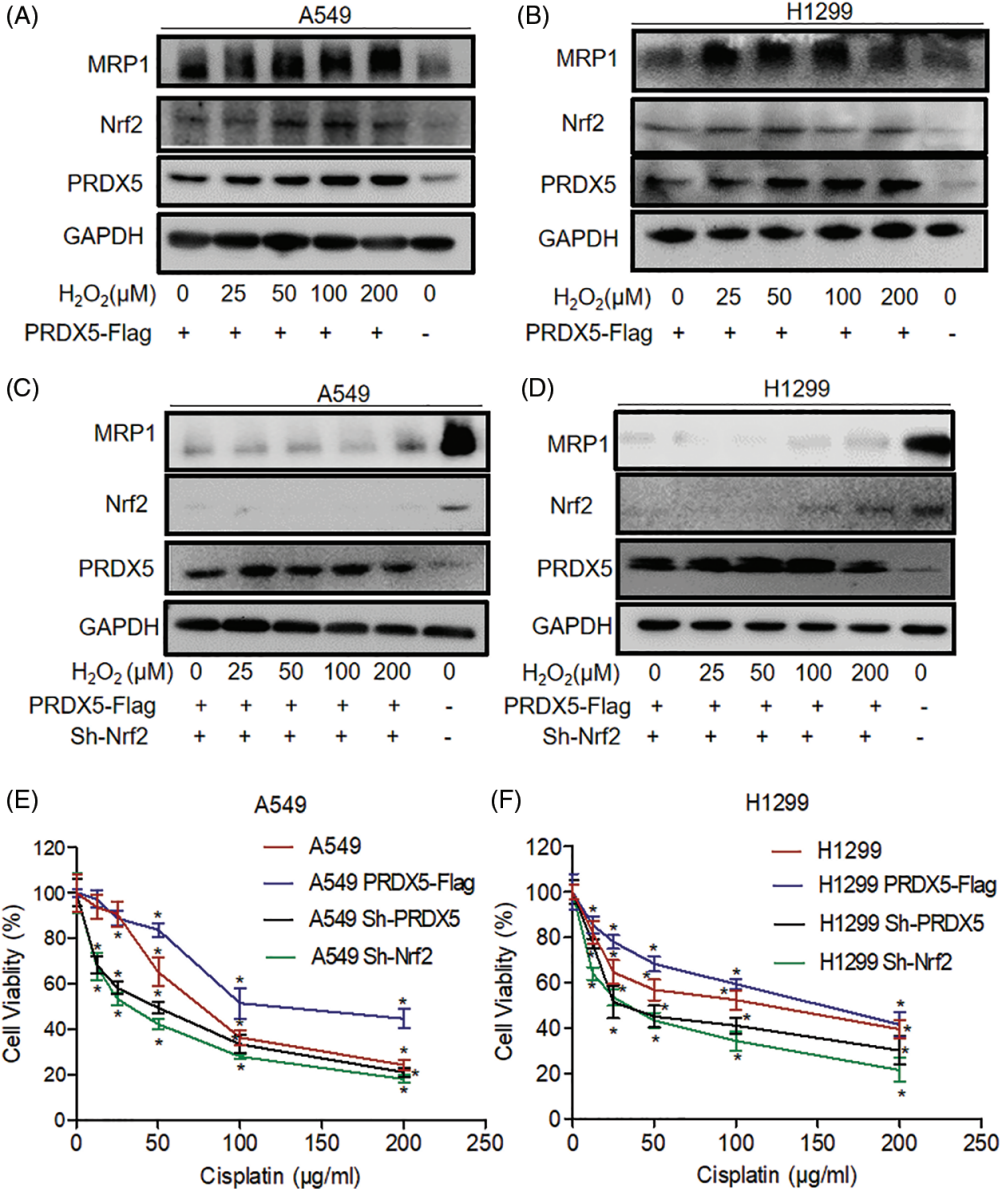
The synergistic effect of PRDX5 and Nrf2 regulates the expression of MRP1 and participates in the resistance of NSCLC cells to cisplatin. (A–B) A549 and H1299 transfected in PRDX5-Flag, and treated with different concentrations (0, 25, 50, 100, 200 μM) of H_2_O_2_ for 24 h. (C–D) A549 and H1299 transfected in PRDX5-Flag or Nrf2 shRNA and treated with different concentrations (0, 25, 50, 100, 200 μM) of H_2_O_2_ for 24 h. We used western blot to detect the expression of MRP1, PRDX5, and Nrf2. GAPDH was measured as a loading control. (E–F) A549 and H1299 cells transfected with PRDX5 shRNA, Nrf2 shRNA, or PRDX5-Flag were treated with different doses (12.5, 25, 50, 100, 200 μg/ml) of Cisplatin for 24 h. MTT assay was used to measure cell viability. The experimental results were expressed as mean ± SD, and the experiment was repeated three times, *p < 0.05.

As shown in Figs. 3A and 3B, the western blot results showed that PRDX5 overexpression significantly enhanced the induction of MRP1 in A549 and H1299 by H_2_O_2_. However, the results in Figs. 3C and 3D indicated that the knockdown of Nrf2 significantly inhibited the expression of MRP1, and overexpression of PRDX5 could not rescue the expression of MRP1. These data suggested that the synergy of PRDX5 and Nrf2 could regulate the expression of MRP1, but Nrf2 had a more direct effect on MRP1.

After showing that the synergy of PRDX and Nrf2 could regulate the expression of MRP1, we used an MTT assay to detect the tolerance of NSCLC cells to cisplatin. The results showed that PRDX5 overexpression could improve the tolerance of A549 and H1299 cells to cisplatin. However, the knockdown of PRDX5 and Nrf2 could significantly reduce the tolerance of NSCLC cells to cisplatin. Moreover, the knockdown of Nrf2 had a more significant impact on NSCLC cells than the knockdown of PRDX5 (Figs. 3E and 3F). This data was also consistent with the results of the western blot.

### The synergy of PRDX5 and Nrf2 induces the proliferation of NSCLC cells in the lung cancer zebrafish model

**Figure 4:**
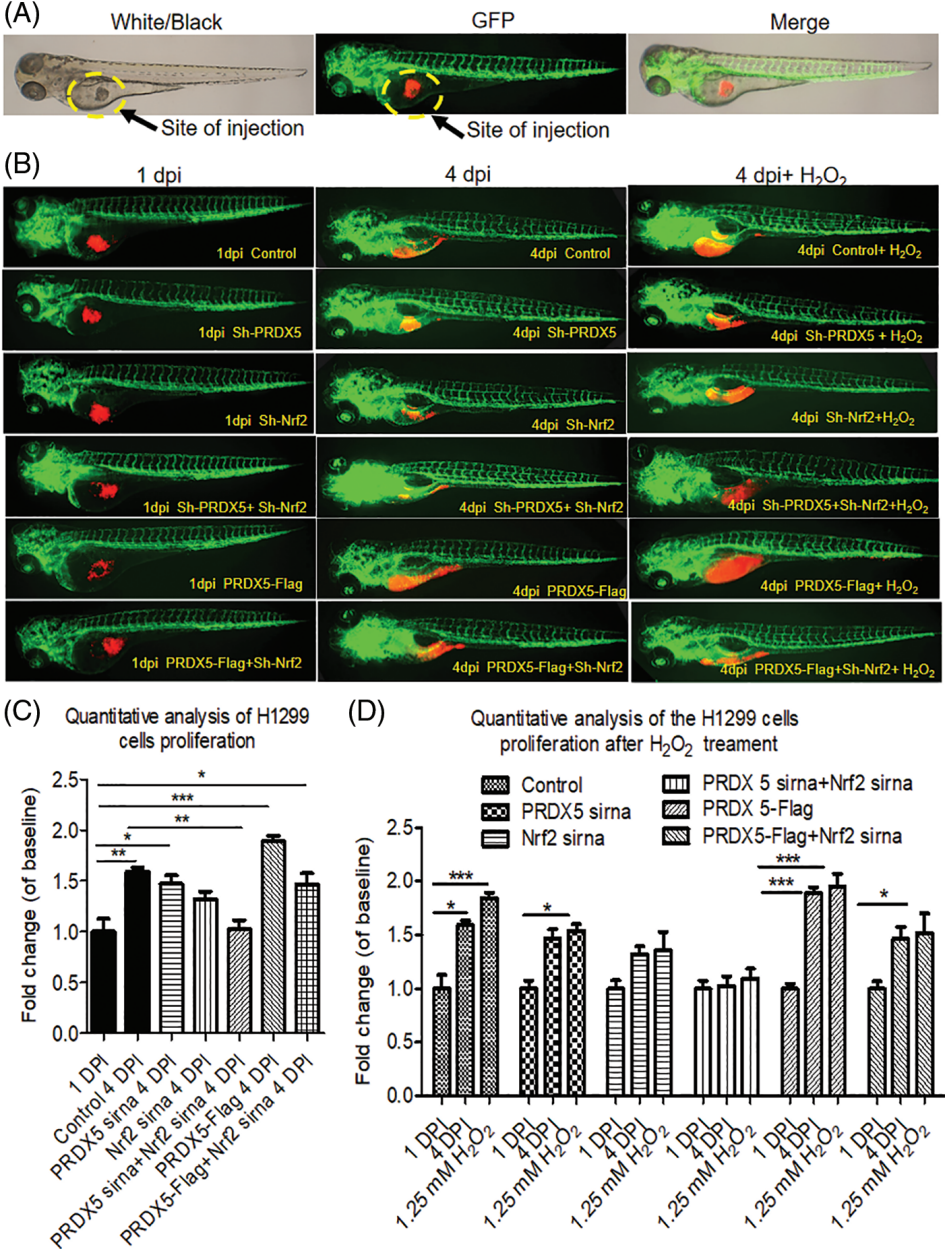
The synergy of PRDX5 and Nrf2 could induce the proliferation of NSCLC cells in zebrafish. (A) Schematic diagram of the injection site. (B) The H1299 cells were transfected with PRDX5-Flag, PRDX5 shRNA, or Nrf2 shRNA. After 24 h, H1299 cells were stained with CM-Dil overnight and collected for injection. Zebrafish embryos injected with H1299 cells without treatment were used as the control group. (C) Statistical analysis of the proliferation rate of NSCLC cells. There were three duplicate holes in each group and 10 embryos per hole. (D) Statistical analysis of the effect of H_2_O_2_ on the proliferation rate of NSCLC cells in zebrafish. There were three duplicate holes in each group and 10 embryos per hole. Significant differences compared with the control group, *p < 0.05, **p < 0.01 and ***p < 0.001.

We used zebrafish embryos as a model to verify the effect of PRDX5 and Nrf2 on the proliferation of NSCLC cells *in vivo*. The results in Fig. 4 showed that PRDX5 shRNA, Nrf2 shRNA, and PRDX5+Nrf2 shRNA could significantly inhibit the proliferation of H1299 cells in zebrafish embryos. Compared with the control group, the knockdown of PRDX5, and the knockdown of Nrf2 and PRX5+Nrf2 could reduce the proliferation rate of NSCLC cells by 0.123, 0.273, and 0.569, respectively. On the contrary, PRDX5 overexpression could increase the proliferation rate of NSCLC cells to 1.997. In addition, PRDX5-Flag+Nrf2 shRNA reduced the proliferation rate of NSCLC cells by 0.128. Meanwhile, H_2_O_2_ could increase the proliferation rate of NSCLC cells in all groups.

### The synergistic effect of PRDX5 and Nrf2 induced the sensitivity of NSCLC cells in the lung cancer zebrafish model to cisplatin

**Figure 5:**
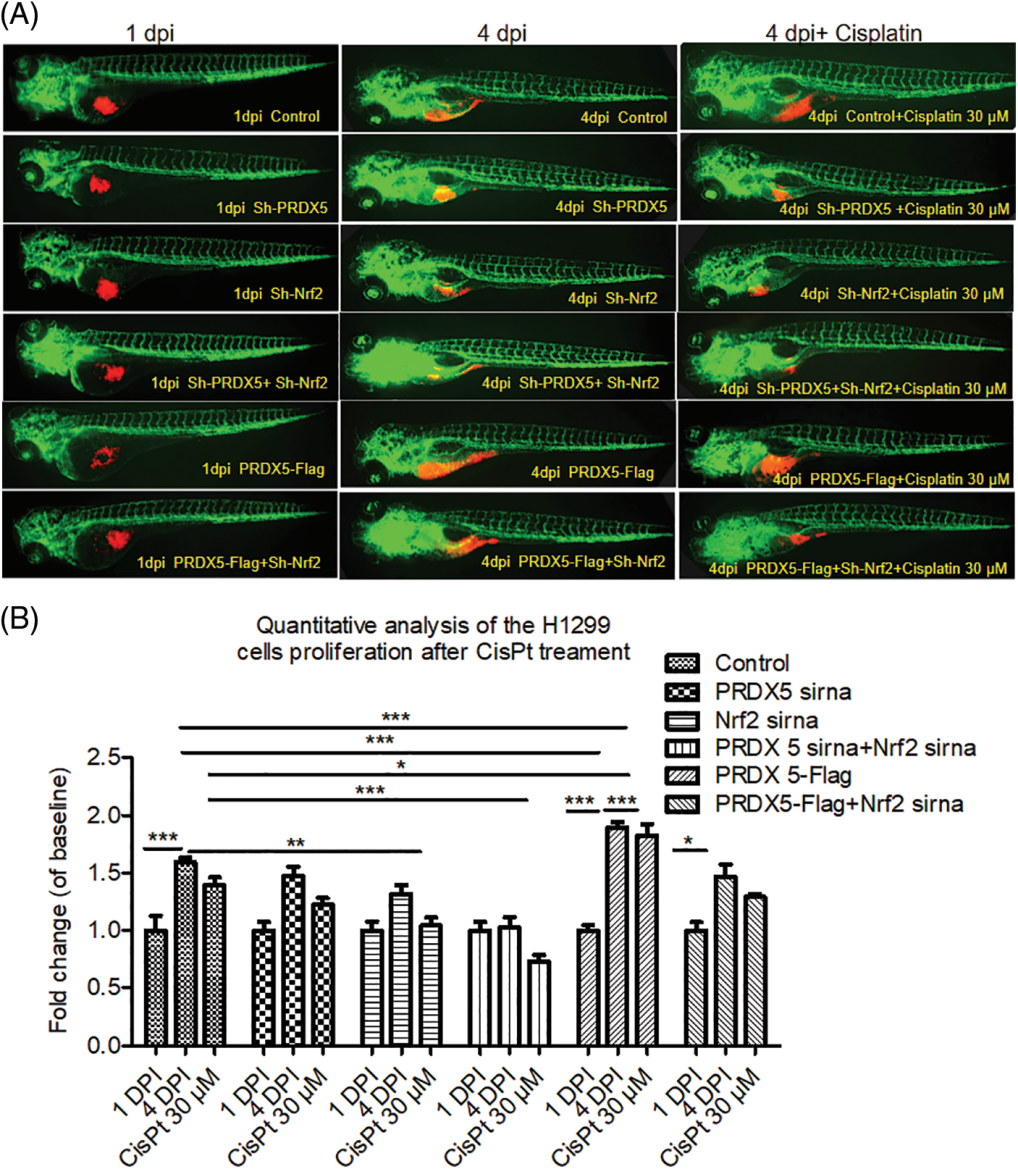
The synergistic effect of PRDX5 and Nrf2 induced the sensitivity of NSCLC cells in zebrafish to cisplatin. (A) The H1299 cells were transfected with PRDX5-Flag, PRDX5 shRNA, or Nrf2 shRNA. After 24 h, H1299 cells were stained with CM-Dil overnight and collected for injection. Zebrafish embryos injected with H1299 cells without treatment were used as the control group. Cisplatin was added to the embryo culture medium on the first day after injection. (B) Statistical analysis of the effects of cisplatin on the proliferation rate of NSCLC cells in zebrafish. There were three duplicate holes in each group and 10 embryos per hole. Significant differences compared with the control group, *p < 0.05, **p < 0.01 and ***p < 0.001.

As shown in Fig. 5, the results showed that cisplatin down-regulated the survival rate of the control group by 0.199. Furthermore, cisplatin down-regulated the survival rate of the NSCLC cells in the PRDX5 shRNA group, Nrf2 shRNA group, and PRDX5+Nrf2 shRNA group by 0.245, 0.273 and 0.295. In addition, cisplatin only reduced the survival rate of 0.067 in the PRDX5 overexpression group. Moreover, the results also showed that the knockdown of Nrf2 could increase the sensitivity of NSCLC cells in zebrafish to cisplatin, and the overexpression of PRDX5 could not reverse it. Combined with the results of our previous detection of MRP1 and MTT analysis, we speculated that the synergistic effect of PRDX5 and Nrf2 increased the expression of MRP1, thereby enhancing the body’s tolerance to cisplatin. Moreover, Nrf2 had a more substantial regulatory effect on MRP1.

### Under oxidative stress, the synergistic effect of PRDX5 and Nrf2 promoted the migration of NSCLC cells in zebrafish

**Figure 6:**
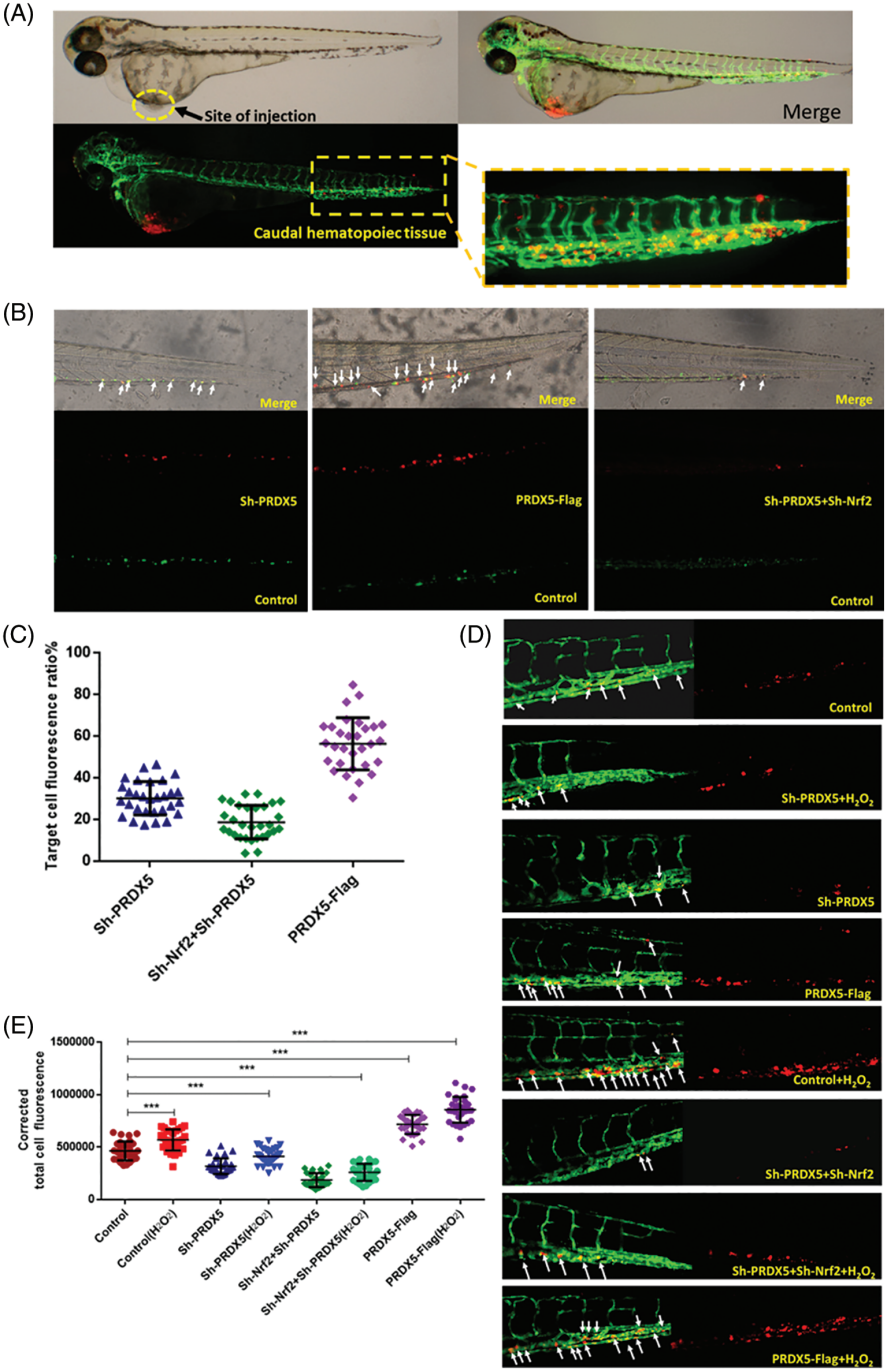
Under oxidative stress, the synergistic effect of PRDX5 and Nrf2 promoted the migration of NSCLC cells in zebrafish. (A) Schematic diagram of injection site and statistical site. (B) H1299 cells in the control group were stained with DIO (green fluorescence). H1299 cells treated with PRDX5 shRNA, Nrf2 shRNA, and PRDX5-Flag were stained with CM-Dil (red fluorescence). The cells with green fluorescence and the target cells with red fluorescence were mixed 1:1 and injected into the perivitelline space of non-fluorescent zebrafish embryos (*Danio rerio*). A fluorescence microscope was used to take pictures on the fourth day after injection (C) for fluorescence intensity measurement. Performed statistical analysis according to the formula (red fluorescence intensity (green fluorescence intensity + red fluorescence intensity)). At least 30 embryo tail fluorescence intensity was counted in each group. (D) PRDX5 shRNA, Nrf2 shRNA, and PRDX5-Flag were transfected into H1299 cells. After 24 h, H1299 was treated with CM-Dil overnight and collected the cells for injection. Observed and photographed with a fluorescence microscope on the fourth day after injection. H_2_O_2_ was added to the embryo culture medium on the first day after injection. (E) Counted the red fluorescence intensity of the tails of at least 30 embryos in each group and made statistics. Significant differences compared with the control group, *p < 0.05, **p < 0.01 and ***p < 0.001.

First, we observed the migration of different groups of cells co-injected into wild-type embryos. H1299 cells without any genetic modification were labeled with green fluorescence, while cells with genetic modification were labeled with red fluorescence. As shown in Fig. 6, the results indicated that the knockout of PRDX5 and PRDX5+Nrf2 significantly reduced the migration efficiency of H1299 cells in zebrafish. The overexpression of PRDX5 promoted the migration efficiency of H1299 cells in zebrafish to 56.63%, which was much higher than the PRDX5 shRNA group (28.8761%) and PRDX5+Nrf2 shRNA group (19.610%).

In addition, we used fluorescent zebrafish to detect the migration of NSCLC cells. The results showed that H_2_O_2_ could increase the number of migrations of cells compared with the control group, and the number of migrations is 1.18 times that of the control group. The knockdown of PRDX5 and PRDX5+Nrf2 inhibited the migration of cells by 0.71 times and 0.33 times, respectively. The overexpression of PRDX5 significantly promoted the migration number of H1299 cells, which was 1.42 times the migration number of the control group (Fig. 6).

## Discussion

Previous studies reported that PRDX5 had an effective antioxidant and scavenging effect on free radicals under oxidative stress [16]. Some research indicated that PRDX5 expression was closely related to cancer cell proliferation, signal transduction, and metastasis [15,40,41]. PRDX5 and Nrf2 are closely associated with oxidative stress, so there are signs that Nrf2 and PRDX5 may have a particular relationship [42,43]. Chen’s study preceded us in demonstrating the existence of the PRDX5-Nrf2 interaction mechanism [27]. In our study, we also found that PRDX5 and Nrf2 were highly expressed in clinical tissue samples of NSCLC patients. Furthermore, with the progress of clinical tumor staging, the expression of PRDX5, Nrf2, and Ki67 was highly positive. Afterward, the co-immunoprecipitation assay results proved that PRDX5 and Nrf2 could combine to form a complex in NSCLC tissues or NSCLC cells. Meanwhile, H_2_O_2_ improved the binding effect of PRDX5 and Nrf2. Our study primarily exploited the visibility of a zebrafish xenograft model to dynamically track changes in NSCLC cell proliferation, metastasis, and drug resistance under altered oxidative stress conditions. We proved that the synergy of PRDX5 and Nrf2 could play an essential role in regulating the proliferation, drug resistance, and mechanism of NSCLC tumors *in vivo* and also consistent with LIOTTA’s research, ROS levels could lead to enhanced tumor cell metastasis [44].

Recent studies have found that the Nrf2-ARE signaling pathway is a crucial negative regulatory system to reduce ROS levels [45,46]. In normal cells and tumor cells, the Keap1-Nrf2/ARE pathway comprises Nrf2 and its inhibitory protein Keap1 (Kelch-like ECH-associated protein 1) and ARE (antioxidant response element) is an important regulatory way to reduce ROS levels [47]. When the cell is subjected to external interference, the cysteine of Keap1 is chemically modified, causing the Keap1-Nrf2 complex. The change of Keap1 causes Nrf2 to quickly enter the nucleus and combine with ARE to activate the antioxidant system [48]. However, many scholars have also pointed out that the abnormal expression of Nrf2 will promote the development of tumors. Singh’s study found that in NSCLC, the up-regulation of Nrf2 expression promotes cell proliferation [49]. Trail M’s research also showed that the abnormal expression of Nrf2 promoted cancer cell proliferation and metastasis [50]. Therefore, the study of non-Keap1 signaling pathways regulated by the direct binding of Nrf2 is significant for the clinical treatment of NSCLC.

When the exogenous overexpression PRDX5 plasmid was transfected into H1299 cells, the expression of Nrf2 also increased. The results indicated that PRDX5, as a newly discovered protein that could bind to Nrf2, could participate in the translational regulation of Nrf2. We also observed that the synergistic effect of PRDX5 and Nrf2 could promote the expression of MRP1 and increase the drug resistance of NSCLC cells. Some scholars found that Nrf2 was positively correlated with the expression of MRP1, and its function was mainly caused by AREs-ARE1 and ARE2 [51]. Our experimental results infer that PRDX5 can indirectly affect MRP1 synthesis by influencing the expression of Nrf2.

In conclusion, PRDX5, a newly discovered molecule that can bind to Nrf2, has a synergistic effect with Nrf2. Meanwhile, PRDX5-Nrf2 significantly regulates NSCLC progression and drug resistance activities in the lung cancer zebrafish models. Oxidative stress can exacerbate this regulation. Furthermore, we will use zebrafish to gene edit PRDX5 or Nrf2 loci and then microinject tumor cells. In more detail, we can understand the signaling pathways regulating tumor cell metastasis and drug resistance mechanism.

## Data Availability

The data supporting this study’s findings are available from the corresponding author upon reasonable request. Correspondence and requests for materials should be addressed to Guoren Zhou, Jinjun Ye, Ruixue Xia, and Hongping Xia.
